# Oscillation dynamics underlie functional switching of NF-κB for B-cell activation

**DOI:** 10.1038/npjsba.2016.24

**Published:** 2016-10-20

**Authors:** Kentaro Inoue, Hisaaki Shinohara, Marcelo Behar, Noriko Yumoto, Gouhei Tanaka, Alexander Hoffmann, Kazuyuki Aihara, Mariko Okada-Hatakeyama

**Affiliations:** 1 Laboratory for Integrated Cellular Systems, RIKEN Center for Integrative Medical Sciences, Yokohama, Japan; 2 Cellular Sensing and Communication Dynamics Research Group, Department of Biomedical Engineering, University of Texas, Austin, TX, USA; 3 Institute of Industrial Science, The University of Tokyo, Tokyo, Japan; 4 Signaling Systems Laboratory, Institute for Quantitative and Computational Biosciences, Department of Microbiology, Immunology, and Molecular Genetics, University of California, Los Angeles, CA, USA

## Abstract

Transcription factor nuclear factor kappa B (NF-κB) shows cooperative switch-like activation followed by prolonged oscillatory nuclear translocation in response to extracellular stimuli. These dynamics are important for activation of the NF-κB transcriptional machinery, however, NF-κB activity regulated by coordinated actions of these dynamics has not been elucidated at the system level. Using a variety of B cells with artificially rewired NF-κB signaling networks, we show that oscillations and switch-like activation of NF-κB can be dissected and that, under some conditions, these two behaviors are separated upon antigen receptor activation. Comprehensive quantitative experiments and mathematical analysis showed that the functional role of switch activation in the NF-κB system is to overcome transient IKK (IκB kinase) activity to amplify nuclear translocation of NF-κB, thereby inducing the prolonged NF-κB oscillatory behavior necessary for target gene expression and B-cell activation.

## Introduction

**Figure 1:**
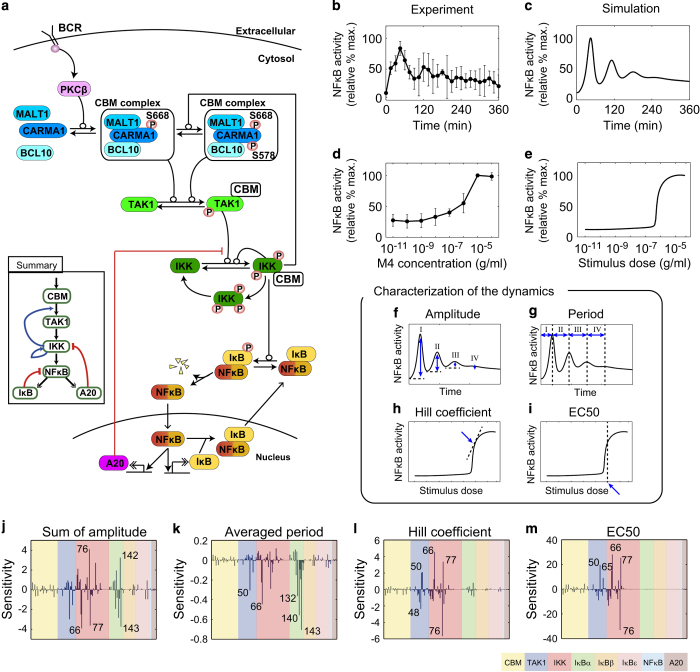
Network structure and sensitivity analysis of nuclear factor kappa B (NF-κB) dynamics. (**a**) Diagram of the BCR-NF-κB signaling network. (**b**–**e**) For NF-κB activity, chicken DT40 B cells were stimulated with anti-IgM mAb (M4) and nuclear NF-κB was assessed by western blotting (Supplementary Figures 3 and 4). (**b** and **c**) Time-course profiles of NF-κB activation after stimulation (M4 concentration; 10^−5^ g/ml). (**d** and **e**) Dose response dynamics of NF-κB activation by BCR stimulation at 45 min. (**b** and **d**) Experimental results. Error bars denote standard error for six independent experiments. (**c** and **e**) Simulation results. (**f**–**i**) Schematic description of characterization of the dynamics. (**f**) Oscillation amplitudes and (**g**) oscillation period in the time-course analysis. (**h**) Hill coefficient and (**i**) EC50 in stimuli dose response analysis. (**j**–**m**) Parameter sensitivity for (**j**) amplitude and (**k**) period of oscillation, (**l**) Hill coefficient and (**m**) EC_50_ for antigen dose response of NF-κB activity. Colors indicate reaction indices for the CBM complex (yellow), TAK1 phosphorylation (blue), IKK phosphorylation (red), IκBα (green), IκBβ (orange), IκBε (pink), NF-κB (light blue) and A20 (brown). Numbers in **j**–**m** indicate the reaction indices in a detailed reaction scheme (Supplementary Figure 1). BCR, B-cell receptor.

Transcription factor nuclear factor kappa B (NF-κB) is activated in response to a variety of extracellular stimuli and regulates transcription of multiple genes involved in cell fate decisions.^1^ Given its central role in many cellular processes, the regulation of this transcription factor is critical in several human diseases.^2–5^ In the canonical signaling pathway in B lymphocytes, NF-κB is activated by B-cell receptor (BCR) signaling via a complex chain of events involving protein kinase C β (PKCβ), CARD containing MAGUK protein1 (CARMA1, also known as CARD11), transforming growth factor (TGF) β-activated kinase 1 (TAK1, also known as MAP3K7) and IκB kinase β (IKKβ). In unstimulated cells, NF-κB is retained in the cytosol in an inactive state due to its association with inhibitors of NF-κB (IκBs). After stimulation, phosphorylation-induced degradation of the IκBs by IKKβ liberates NF-κB for nuclear translocation, where it regulates transcription of target genes (Figure 1a, Supplementary Section 1). NF-κB activity is further regulated by multiple feedback loops in the network, resulting in complex dynamical activity profiles.^6–13^

NF-κB activity exhibits two characteristic behaviors, oscillations and switch-like activation, regulated by negative and positive feedback, respectively. Prolonged oscillatory nuclear translocation of NF-κB, whose dynamics are important for induction of gene expression, has been documented in a variety of cells stimulated with ligands such as tumor necrosis factor (TNF).^8,11,14,15^ NF-κB also shows cooperative switch-like activation dynamics in response to an increased dosage of TNF ligand at the single cell level^11^ and in bulk populations of BCR-activated B cells.^13^ It is known that interlinked positive feedback loops associated with negative feedback loops are the basis of sustained periodic oscillations in the cell cycle and in circadian rhythms.^16–19^ However, unlike these examples, positive feedback loops identified in the NF-κB signaling network do not induce those types of NF-κB oscillations, but instead induce damped oscillations. These observations suggest that the positive feedback loops embedded in the NF-κB system have different biological functions from other well-studied biological oscillators. In particular, it is unclear whether switch-like activation and oscillations are separate properties emerging from different regulatory mechanisms in the network, or represent two sides of the same coin.

In this report, we expand our previous mathematical model^13^ to include the BCR signaling pathway and transcriptional regulation of NF-κB to analyze the dynamic behaviors of NF-κB in B cells stimulated with extracellular surrogate antigen. We show using the model and quantitative experiments followed by validation analysis that NF-κB activity in B cells exhibits oscillations after switch-like activation. Using a variety of B cells with artificially modified NF-κB signaling networks, oscillations and switch-like activation of NF-κB could be dissected and, in some conditions, these two behaviors were separated. We show that switch-like activation caused by positive feedback loops is essential to compensate for the transient nature of upstream IKK activity and to induce amplified accumulation of nuclear NF-κB, which is in turn necessary for inducing subsequent oscillations for target gene expression.

## Results

### A comprehensive mathematical model for the NF-κB network

To investigate the roles and regulatory mechanisms of feedback loops for NF-κB activation dynamics in BCR signaling, we first constructed a comprehensive mathematical model by integrating earlier models for BCR signaling^13^ and transcriptional regulation of NF-κB^9^ (Figure 1a). Our experimental results (Figure 1b) revealed an oscillatory response reminiscent of that seen with TNF,^6^ an observation suggesting that the NF-κB-IκB system may be regulated similarly in both BCR and TNF signaling networks. We then extended the models, incorporating details of the formation of the CARMA1, B cell chronic lymphocytic leukemia 10 (BCL10), and/or mucosa-associated lymphoid tissue (MALT) lymphoma translocation gene 1 (MALT1) (CBM signalosome) complex, which has critical roles in regulating BCR signaling. Adaptor proteins BCL10, MALT1 and CARMA1, which were implicitly considered but not included in our previous model,^13^ were newly added in the current model to examine and validate the effects of those molecules on NF-κB dynamics. The current model includes multiple feedback loops. Two positive feedback loops are operative in a phosphorylation cascade for TAK1 and IKKβ; one is the enhanced TAK1 activation mediated by IKKβ-dependent CARMA1 phosphorylation at serine 578 (S578),^20^ and the other is introduced by *trans* auto-phosphorylation of IKKβ.^21^ The former positive feedback loop reactivates and sustains TAK1 activity upon second phosphorylation of CARMA1 at S578 after the first PKCβ-dependent CARMA1 S668 phosphorylation.^13^ These positive feedback regulations contribute to the switch-like activation of IKKβ and NF-κB. In addition, there are three transcriptionally inducible negative feedback loops located in the downstream region of the network. Signal-activated NF-κB translocates into the nucleus and initiates transcription of a variety of genes, including those encoding IκBα and IκBε, which force NF-κB into an inactive state in the cytosol. TNF alpha-induced protein 3 (A20, also known as TNFAIP3) is also induced upon NF-κB activation and negatively regulates IKKβ activity.^22,23^ Those negative feedback loops are thought to be important for induction of the oscillatory response of NF-κB. Model parameters were optimized to recapitulate experimental observations obtained using chicken DT40 B cells stimulated with anti-IgM (mAb M4; details of the model are described in Supplementary Figures 1–4, Supplementary Tables 1 and 2, Supplementary Section 2). Numerical simulations using the comprehensive model reproduced the time course and dose response properties of signaling activities of TAK1, IKKβ and PKCβ-triggered CARMA1 phosphorylation at S668 in the short-term time course in a variety of conditions in our previous experiments^13^ (Supplementary Figure 5), as well as oscillatory behavior observed on longer time scales (Figure 1c). The Hill coefficient, which indicates cooperativity of the network, and EC_50_, the stimuli dosage inducing half-maximal activity (representing threshold values for activation) for NF-κB activation obtained by experiment and simulation were 2.68±6.92 and 1.48×10^−6^±1.37×10^−6^ g/ml, 3.49±0.168 and 6.07×10^−7^±1.04×10^−8^ g/ml, respectively (Figures 1d and e). Furthermore, the simulation recapitulated the response in BCL10 and CARMA1^S578A^ mutants and wild-type cells in the presence of PKC and IKKβ inhibitions in our earlier study^13^ (Supplementary Figure 5, Supplementary Table 3). Thus, this comprehensive model was considered suitable for further theoretical analyses.

### Critical parameters responsible for switch-like and oscillatory responses of NF-κB

To identify reactions critical for oscillations and switch-like responses of NF-κB activity, we performed a numerical sensitivity analysis for amplitudes and periods of oscillations, as well as Hill coefficient and EC_50_ of the antigen dose response (Figures 1f–i, Supplementary Table 4; see numerical definitions of the dynamics in the Materials and Methods' section). In what follows, numbers in parentheses indicate reaction indices (Supplementary Figure 1). We observed that the total sum of oscillation amplitudes was highly sensitive to perturbations of reactions involving IKKβ (66, 76, 77) and IκBα (142, 143; Figure 1j). The oscillation period was more sensitive to TAK1 (50), IKKβ (66) and IκBα (132, 140, 143; Figure 1k). Trends of parameter sensitivities for the sum of amplitudes and averaged period of NF-κB oscillations (Figures 1j and k) were generally consistent with those analyzed for individual peaks in the oscillations (Supplementary Figure 6). For the Hill coefficient and EC_50_ values of NF-κB activity, highly sensitive parameters were mostly enriched in the upstream signaling reactions for TAK1 and IKKβ (48, 50, 65, 66, 76, 77; Figures 1l and m). These results show that reactions involved in positive feedback loops are strongly associated with oscillation amplitudes and periods. It is noteworthy that, although the switch-like induction of the first peak of nuclear NF-κB may significantly affect subsequent oscillation, the two dynamical behaviors are independently regulated by different components of the pathway.

### Inhibition and enhancement of feedback loops

**Figure 2:**
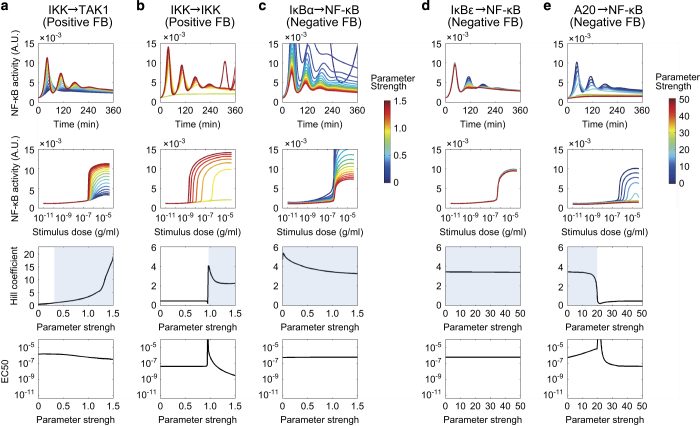
Individual effects of feedback loops in nuclear factor kappa B (NF-κB) dynamics. Time course (upper panels), antigen dose response (second panels), Hill coefficient (third panels) and EC_50_ (bottom panels) of NF-κB activity in response to changes in reaction rate of the feedback (FB) loop; (**a**) Positive feedback loop from IKKβ to TAK1 (49, *k*_*TpCIKK3*_). (**b**) Auto-positive feedback loop in IKKβ (76, *k*_*IpCfaIKKppC*_). (**c**) Negative feedback loop mediated by NF-κB-induced IκBα expression (141, *k*_*prodmrnaikba*_). (**d**) Negative feedback loop mediated by NF-κB-induced IκBε expression (152, *k*_*prodmrnaikbe*_). (**e**) Negative feedback loop mediated by NF-κB-induced A20 expression (158, *k*_*prodmrnaa20*_). Parameter strength is shown as fold-change values in color (high; red, low; blue) compared with the original parameter value (Supplementary Table 1). For the Hill coefficient, parameters showing switch-like response (Hill coefficient >1) are in blue. Numbers and kinetic rate constants shown in brackets above indicate reaction indices and changed parameters in Supplementary Figure 1 and Supplementary Table 1, respectively.

The NF-κB network contains five feedback regulatory loops, two positive and three negative (Figure 2). The positive feedback loops are fully contained in the upstream part of the network, whereas the negative feedback loops operate at the level or downstream of NF-κB (IκB’s), or linking downstream and upstream network components (A20). It is known that the negative feedback loops mediated by transcriptional induction of IκBα and IκBε have distinct roles in controlling oscillation dynamics.^6,9,24^ Consistent with previously reported results, we observed that changes in the parameters controlling NF-κB-induced IκBα expression (141) significantly altered oscillation amplitudes and periods (Figure 2c). Changes in the parameter of NF-κB-induced IκBε expression (152) altered oscillation dynamics only at the late phase of the response (Figure 2d). We also determined that these negative feedback regulators had small effects on the Hill coefficient and EC_50_ of NF-κB peak activity (Figures 2c and d). On the other hand, the positive feedback loops operating upstream of NF-κB had more global effects. A stronger positive feedback from IKKβ to TAK1 (49) increased the amplitude of oscillations and the steepness of the switch responses (Figure 2a). Changes in the parameter of the autoregulation of IKKβ (76) dramatically altered the oscillatory dynamics of NF-κB signals as well as the overall shape of the stimulus dose response (Figure 2b). The negative feedback loop consisting of NF-κB-induced A20 expression (158) affected the Hill coefficient and EC_50_, as well as oscillation amplitudes and periods (Figure 2e).

**Figure 3:**
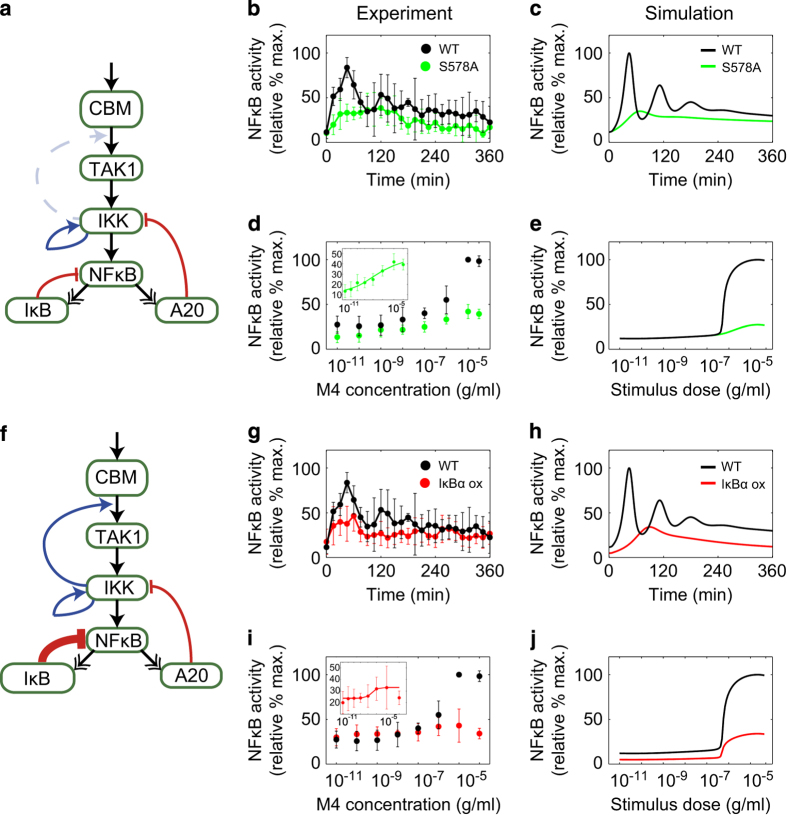
Experimental validation of feedbacks for oscillation and cooperativity in nuclear factor kappa B (NF-κB) activity. (**a**) The positive feedback loop from IKKβ to TAK1 (reaction index 49, aqua line) was eliminated by CARMA1^S578A^. (**b**–**e**) Experimental and simulation results of time-course analysis (M4 concentration; 10^−5^ g/ml) and dose response analysis at depicted concentrations in CARMA1^S578A^ cells (S578A, green) and wild-type cells (WT, black) at 45 min after stimulation. (**b** and **d**) Experimental results. (**c** and **e**) Simulation results. (**f**) The negative feedback loop mediated by NF-κB-induced IκB expression (reaction index 140, bold red line) was enhanced by IκBα overexpression (IκBα ox). Experimental and simulation results of time course (**g** and **h**) and dose response (**i** and **j**) analysis. IκBα ox (red) and wild type (WT, black). Error bars denote standard error for five and four independent experiments in CARMA1^S578A^ cells and IκBα ox cells, respectively. Representative blot images can be found in Supplementary Figures 3 and 4. The inset in the dose response experiments shows a fitting curve by the Hill equation.

The above analysis predicted that the positive feedback loop from IKKβ to TAK1 would affect oscillation dynamics and the Hill coefficient (switch) but not the EC_50_ (threshold) of the dose response of NF-κB activity (Figure 2a). On the other hand, it was also predicted that changes in IκBα-related reactions would alter the oscillation patterns but not the switch-like response of NF-κB (Figures 1j–m). To test these predictions experimentally, we measured the time course and dose response of NF-κB activity using DT40 cells with either a CARMA1 S578 mutation to alanine (CARMA1^S578A^), which mimics the absence of positive feedback from IKKβ to TAK1, or IκBα overexpression (IκBα ox), which mimics strong negative IκBα feedback (Figure 3, Supplementary Figures 3 and 4, Supplementary Table 3). As predicted, we found that in the CARMA1^S578A^ mutation, both oscillations and switch-like responses became indistinct (Figures 3a–e). The Hill coefficient of the CARMA1^S578A^ mutant (experiment; 0.286±0.064, simulation; 0.530±0.028) was lower compared with that in the wild-type cells, whereas the EC_50_ did not change significantly (experiment; 3.74×10^−7^±4.44×10^−7^ g/ml, simulation; 1.51×10^−6^±2.55×10^−7^ g/ml; Figure 2a). In the IκBα overexpressing cells, the oscillations became indistinct, however, the steepness of the dose response did not change significantly (Figures 3f–j). The Hill coefficient and EC_50_ values obtained by experiment (1.46±0.204 and 2.57×10^−7^±4.21×10^−8^ g/ml) and simulation (2.56±0.124 and 6.69×10^−7^±1.47×10^−8^ g/ml) for IκBα overexpression were not significantly different from those in wild-type cells. These observations indicate that oscillations and switch-like activation in the NF-κB system do not necessarily have to occur together, and can be separated.

### Changes in protein abundance decouple oscillatory and switch-like responses

**Figure 4:**
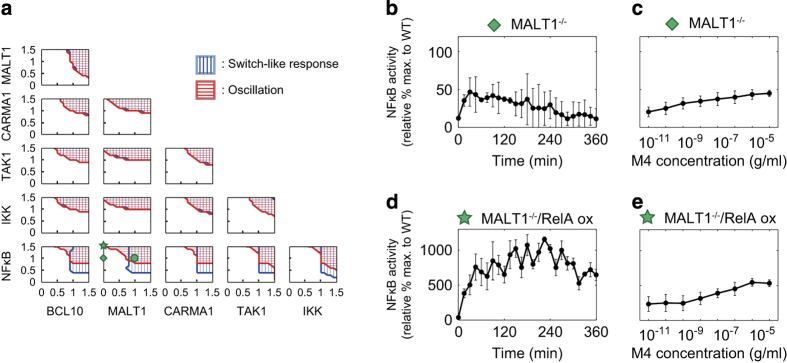
Effects of nuclear factor kappa B (NF-κB) abundance on decoupling of oscillatory and switch-like dynamics. (**a**) Theoretically obtained two-parameter regions of the indicated protein abundance combinations (BCL10, MALT1, CARMA1, TAK1, IKK, and NF-κB) for oscillations and switch-like responses. Red and blue regions indicate oscillations and switch-like responses, respectively. Values on the vertical and horizontal axes indicate relative abundances of the proteins. The green circle indicates original values in the model. (**b**–**e**) Experimental validation. (**b** and **c**) MALT1-deficient (MALT1^−/−^, shown as the green diamond in **a**) and (**d** and **e**) NF-κB (RelA)-forced expression in MALT1-deficient cells (MALT1^−/−^/RelA ox, shown as the green star in **a**). (**b** and **d**) Time courses after M4 (10^−5^ g/ml) stimulation. (**c** and **e**) Dose responses. Representative blot images can be found in Supplementary Figures 3 and 4.

To test the hypothesis that oscillations and switch-like responses can be separated, we next investigated the combinational effect of abundance of two proteins in the signaling pathway on the dynamics of NF-κB (Figure 4a; see numerical definitions of the dynamics in the 'Materials and Methods' section). Our results showed that most combinations of two proteins among BCL10, MALT1, CARMA1, TAK1, IKKβ and NF-κB result in both oscillations (red) and switch-like responses (blue) (Figure 4a, details are shown in Supplementary Figures 7 and 8). A notable exception to this trend was NF-κB. Our analysis predicts that a low abundance of NF-κB results in switch-like responses but not oscillations in some parameter regions, whereas high NF-κB abundance alone is likely to be sufficient to produce oscillations in other parameter regions. Specifically, the computational analysis predicted that cells with low MALT1 and high NF-κB abundances would show decoupling of oscillations and switch-like responses (Supplementary Figure 7). To confirm this experimentally, we determined NF-κB activity using MALT1 knockout (MALT1^−/−^) cells, with or without NF-κB (RelA) overexpression (RelA ox; Figures 4b–e, Supplementary Figures 3 and 4). We observed NF-κB oscillations but not switch-like responses (Hill coefficient: 0.458±0.043, EC_50_: 1.41×10^−7^±3.29×10^−8^ g/ml) in the MALT1^−/−^/RelA ox mutant cells (Figures 4d and e). The MALT1^−/−^ mutant cells exhibited neither of the responses (Hill coefficient: 0.118±1.19×10^−25^, EC_50_: 8.99×10^−14^±1.53×10^−14^ g/ml; Figures 4b and c).

### Increased NF-κB abundance compensates for reduced upstream signaling activity

**Figure 5:**
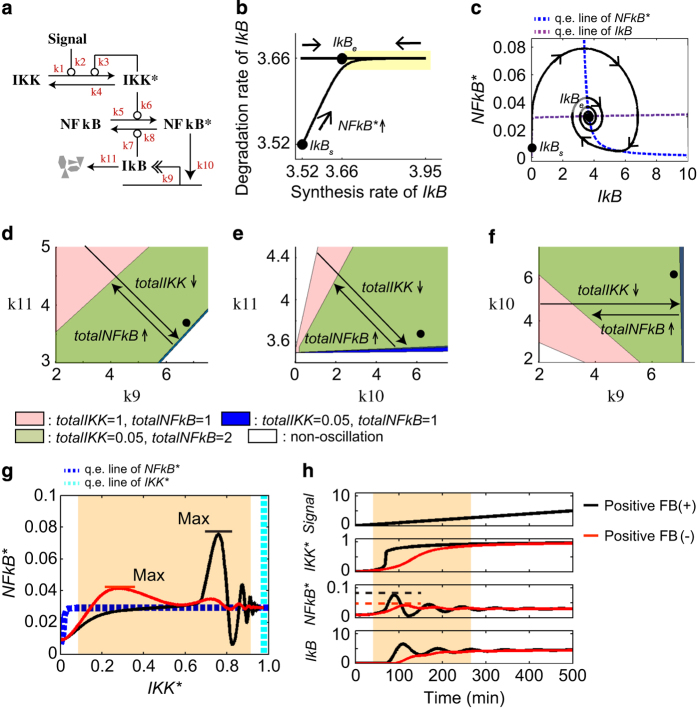
Quasi-equilibrium (q.e.) analysis of positive feedback regulation. (**a**) Schematic illustration of the core model. Asterisks indicate active forms. (**b**) Synthesis and degradation rates of *IkB*. (**c**) Model trajectory in an NFkB*-*IkB* plane. (**b** and **c**) *IkBs* and *IkBe* show equilibrium points before and after B-cell receptor (BCR) stimulation, respectively. (**d**–**f**) Oscillation regions with respect to synthesis and degradation rate constants of *IkB* upon changes of *totalIKK* and *totalNFkB* in the equilibrium state. (**d**) Basal synthesis rate constant *k*_*9*_ and degradation rate constant *k*_*11*_. (**e**) NFkB* induction rate constant *k*_*10*_ and degradation rate constant *k*_*11*_. (**f**) Basal synthesis rate constant *k*_*9*_ and NFkB* induction rate constant *k*_*10*_. (**g**) The q.e. line in signal=10 and the model trajectories in the core model with and without the positive feedback loop. (**h**) Time course presentation of **g**. NF-κB, nuclear factor kappa B.

Why do changes in NF-κB abundance cause oscillations in the absence of switch-like responses? To answer this question, we constructed a core model consisting of only core components, IKKβ, NF-κB and IκBα (Figure 5a, Supplementary Section 3), which are associated with critical parameters responsible for oscillations and switch-like responses (Figures 1j–m). Using this model, we performed equilibrium and quasi-equilibrium (also called nullcline) analysis (Supplementary Figure 9), which allowed us to qualitatively infer the type of dynamics expected near the equilibrium point.

The quasi-equilibrium analysis showed that oscillations are induced when the synthesis rate of the negative regulator *IkB* is transiently faster than its maximum degradation rate constant *k*_*11*_, which corresponds to *IkBe* in Figures 5b and c. Only an increase in NFkB* can overcome the degradation rate of *IkB* (yellow oscillation range, Figure 5b). An increase in *totalNFkB* causes an increase in NFkB* even if IKK* is low. Therefore, an increase in NF-κB abundance can induce adequate synthesis of IκB beyond its maximum degradation rate, even in spite of weak upstream signals, resulting in oscillations of NF-κB (black circles and green regions in Figures 5d–f). On the other hand, a decrease in *totalIKK* causes a major decrease in IKK* when the threshold is not exceeded (Supplementary Figure 9e), resulting in decreased or absent NFkB* oscillations (black circles and indigo regions in Figures 5d–f).

### Roles of positive feedback in NF-κB oscillation dynamics and target gene expression

We showed that the two positive feedback loops (IKKβ-TAK1 and IKKβ autoregulation) control the dynamics of NF-κB activity (Figures 2a, b and 3a–e, and Supplementary Figures 5m–o). Yet, protein abundance (Figure 4), equilibrium and quasi-equilibrium analyses (Figure 5) showed that switch-like activation of NF-κB is not absolutely necessary for induction of oscillations. In that case, what is the fundamental role of positive feedback regulations in the NF-κB system? To answer this question, we next analyzed the model trajectories in the phase plane corresponding to the core model in the absence or presence of positive feedback. The analysis showed that the model trajectory developed stronger NF-κB fluctuation when approaching equilibrium in the presence of feedback (black line) compared with a model without feedback (red line; Figure 5g). The time-course analysis showed that the first oscillation amplitude of NFkB* was much faster and higher in the presence of positive feedback and it was subsequently followed by prolonged oscillations (Figure 5h). IKK* also showed rapid velocity in the presence of feedback. This analysis demonstrated that the positive feedback loops enable prolonged oscillations of NF-κB activity by increasing the initial velocity of IKKβ and the amplitude of nuclear NF-κB.

**Figure 6:**
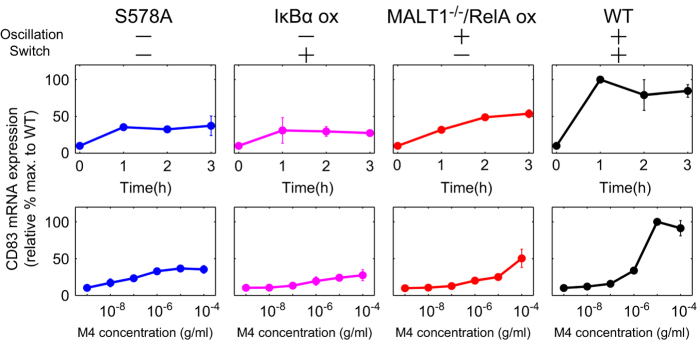
Gene expression in the presence and/or absence of oscillation and switch activation. Time-course analysis (upper panels, M4 concentration; 10^−5^ g/ml) and dose response analysis at 1 h after stimulation (bottom panels; *N*=2) in CARMA1^S578A^ mutant cells (S578A), IκBα overexpression cells (IκBα ox), MALT1-deficient cells with NF-κB (RelA) overexpression (MALT1^−/−^/RelA ox) and wild-type cells (WT). The values of *CD83* mRNA expression are displayed as the relative % maximum of wild-type cells. NF-κB, nuclear factor kappa B.

Prolonged oscillations in NF-κB activity are important for induction of target genes.^8,11,14^ Therefore, we next elucidated the effect of the two responses of NF-κB dynamics on mRNA time course and dose response of *CD83*, a representative NF-κB target gene involved in B-cell maturation.^25^ We tested four cell conditions; CARMA1^S578A^ mutant cells (no oscillation or switch response), IκBα overexpression cells (no oscillation but switch response), MALT1 mutant cells with NF-κB (RelA) overexpression (oscillation but no switch response) and wild-type cells (both oscillation and switch response; Figure 6). The *CD83* gene expression level in the mutant cells was generally lower than in the wild-type cells, even though the NF-κB activity in MALT1^−/−^/RelA ox cells was higher than in the wild-type cells (Figure 4d). This NF-κB overexpressing mutant, which is not associated with the switch response, also showed a slight increase in *CD83* gene expression compared with the other mutants, implying that NF-κB oscillations themselves may have a small effect on NF-κB target gene expression. The wild-type cells also had a strong, prolonged and steep dose increase in *CD83* gene expression. Interestingly, the Hill coefficient and EC_50_ for *CD83* gene expression in the wild-type cells was 8.19±5.35×10^−16^ and 1.14×10^−6^±2.93×10^−8^ g/ml (Supplementary Table 6), respectively, suggesting that cooperative threshold properties of the NF-κB network are reflected in mRNA expression levels.

## Discussion

Positive feedback regulation has several functional roles in biological systems: to sustain molecular activity, to trigger oscillation or to assign a threshold, as seen, for example, in the MAPK and Cdc2/APC networks.^19,26^ In the NF-κB network, the most important role of the positive feedback loops is to amplify the peak maxima of NF-κB nuclear translocation, which can induce the prolonged NF-κB activity necessary for target gene expression. Although two positive feedback loops are operative in the NF-κB signaling pathway, they do not provide sustained IKKβ activity to create prolonged NF-κB oscillation (Supplementary Figure 5d). Because the upstream IKK activity is transient, only increased NF-κB nuclear abundance can compensate for weak upstream IKK activity. Thus, the resulting high-amplitude NF-κB oscillations solely drive productive transcriptional responses important for activation of B cells.

As a result of the current analysis, several key features were clarified as unique properties of the NF-κB network. First of all, switch-like activation and oscillatory behaviors of NF-κB are independently controllable through changes in kinetic parameters and abundance of proteins in the signaling network. Earlier studies indicated that NF-κB oscillation dynamics are mainly controlled by the IKK-IκB negative feedback loop.^6^ However, our analysis showed that NF-κB oscillation is also controlled by stoichiometry and parameters associated with adaptor proteins such as CARMA1 and MALT1, molecules whose mutation and overexpression are responsible for the pathogenesis of some cases of B-cell lymphoma.^2,27,28^ Furthermore, although trends of parameter sensitivities in the NF-κB network are quite similar for all oscillation peak amplitudes, the sensitivity values are increased by peak after the peak (Supplementary Figures 6a–d). This result suggests that oscillation amplitude, particularly in the late phase of oscillations, is more likely to be affected by small changes or noises, such as in external ligand concentrations and protein abundances in individual cells.

Our analysis provides rational evidence showing that NF-κB activity is under tight control of kinetics and abundance of proteins in the signaling pathway, and that dysregulation of this control can lead to abnormal NF-κB activation. In addition, switch-like expression of the *CD83* gene suggests that the NF-κB network is a multifaceted, highly cooperative system for cellular commitment of B cells. Although we could not completely exclude the possibility of species differences, earlier studies strongly suggested that BCR signaling in chicken DT40 B cells can recapitulate, at least, the NF-κB activation pathway and its dynamics in primary mouse B cells;^13,29,30^ therefore, our current study may shed light on the general mechanisms of gene expression regulated by NF-κB during B-cell differentiation.

In a theoretical aspect, because NF-κB shows damped oscillations, our mathematical analysis is different from the usual bifurcation analysis for attractors like sustained oscillations and switching phenomena between different periodic oscillations. So far, many mathematical studies on oscillatory dynamics in biological systems, such as cell cycle and circadian rhythms, have focused on sustained oscillations,^17,31^ whose qualitative changes are tractable in terms of the conventional bifurcation theory. However, the mathematical formulation of qualitative changes in transient dynamics as presented in this study is highly significant as well, because such dynamics are responsible for the functionality of biological systems.

## Materials and Methods

### Generation of mutants and transfectants of DT40 cells

Chicken RelA and IκBα cDNA were generated by PCR. Each cDNA was cloned into the pApuro expression vector.^29^ These constructs were transfected into MALT1-deficient^13^ or wild-type DT40 cells by electroporation as described elsewhere.^29^ Genomic clones of chicken *RelA* were obtained by PCR using oligonucleotides designed on the basis of NCBI database sequences (RelA; NM_205129) as primers and DT40 genomic DNA as a template. CARMA1 (wild type and its S578A mutant) and monomeric EGFP tagged wild-type RelA knock-in vectors were constructed as described previously^20^ to generate a mini gene in the respective endogenous locus. Wild-type and various mutant DT40 cells were cultured in RPMI 1640 (GIBCO BRL, Carlsbad, CA, USA and Invitrogen, Carlsbad, CA, USA) supplemented with 10% fetal bovine serum, 1% chicken serum, 50 μ mol/l 2-ME (Wako), 4 m mol/l l-glutamine and antibiotics.

### Reagents

RelA antibodies (Abs) were purchased from Cell Signaling Technology (Darmstadt, Germany) and Merck Millipore (Darmstadt, Germany). The anti-chicken IgM mAb, M4,^29^ was used for BCR stimulation.

### Cell fractionation and western blots

For NF-κB activity, nuclear and cytoplasmic fractions were prepared as described previously.^13^ In brief, the cells were lysed with lysis buffer containing 50 m mol/l Tris-HCl (pH 7.5), 0.5% Triton X-100, 137.5 m mol/l NaCl, 10% glycerol, 5 m mol/l EDTA and a proteinase inhibitor cocktail (Roche, Basel, Switzerland). The nuclei were separated by centrifugation and the pellets were resuspended in 1% NP-40 lysis buffer and used as the nuclear fraction.

Western blot analysis was carried out after stimulation with anti-IgM (M4) as described previously.^13,20,29^ The samples were subjected to western blot, and RelA activity was quantified from the intensities of protein bands using a Multi Gauge version2.2 (Fujifilm, Tokyo, Japan) densitometer.^13^ For protein detection, the ECL Plex fluorescent western blotting system and ImageQuant LAS 4000 (GE Healthcare, Little Chalfont, UK) were used.

### Gene expression analysis

Total RNA was purified using NucleoSpin RNA (MACHEREY-NAGEL GmbH & Co., Duren, Germany) and subjected to cDNA synthesis and quantitative PCR using the KOD SYBR qPCR set (TOYOBO Life Science, Tokyo, Japan) according to the manufacturer’s instructions. To detect *CD83* mRNA expression, the following primers yielding a 228-bp PCR product were used: sense; 5′- cttcagcagcctacactctactcttcac-3′; antisense: 5′- ggatgacgagattccacatcaaggac-3′.

### Mathematical model

A comprehensive diagram of the BCR-activated NF-κB signaling network is shown in Supplementary Figure 1. The mathematical model was built on the basis of 47 ordinary differential equations with 196 parameters. Details about the rate laws and parameter values can be found in Supplementary Table 1. Steady state values in resting cells were obtained to simulate the basal level (initial values) of the cell before BCR stimulation. Then, computer simulation was performed using the function for the input signal shown in Supplementary Figure 2a. Simulation was carried out with the function *CVODE* (http://computation.llnl.gov/casc/sundials/main.html) in C language. Parameter values were searched by genetic algorithm with Unimodal Normal Distribution Crossover^32^ and Minimal Generation Gap.^33^ In genetic algorithm, we used the following cosine similarity as fitness function.

$$\mathrm{CS}=\overset{n}{\underset{i=1}{\sum }}w_{i}\left(1-{\left(\frac{{\overset{\to }{x}}_{simulation,i}\cdot {\overset{\to }{x}}_{experiment,i}}{\left|{\overset{\to }{x}}_{simulation,i}\right|\left|{\overset{\to }{x}}_{experiment,i}\right|}\right)}^{2}\right),$$

where CS is cosine similarity, *n* is the number of molecules (CARMA1, TAK1, IKK or NF-κB), *w*_*i*_ is a weight parameter, ${\overset{\to }{x}}_{simulation,i}$ is vector for values of the molecule *i* at each time point in the simulation, ${\overset{\to }{x}}_{experiment,i}$ is vector for activities of the molecule *i* at each experimental time point, ${\overset{\to }{x}}_{simulation,i}\cdot {\overset{\to }{x}}_{experiment,i}$ is the inner product of ${\overset{\to }{x}}_{simulation,i}$ and ${\overset{\to }{x}}_{experiment,i}$, and |⋅| denotes the magnitude of a vector. A low cosine similarity means that dynamic behaviors of the molecules in simulation are similar to the ones in experiment. We used the parameter set with the lowest cosine similarity in our parameter search.

### Definition of amplitudes and periods of oscillation

Quantitative measures for amplitudes and periods of oscillation of NF-κB (Figures 1f and g) were defined as follows: the first peak amplitude is defined as the difference between the height of the first peak and the basal value before cell stimulation. The second amplitude is defined as the difference between the height of the second peak and the value of the bottom after the first peak. The third and fourth amplitudes were defined in the same way as described above. Periods were defined as the time lengths between the peaks.

### Hill equation

The Hill equation was defined as follows:

$$y=bottom+(top-bottom)\frac{x^{h}}{EC50^{h}+x^{h}},$$

where 'bottom' is minimum activity, 'top' is maximum activity, EC50 is half-maximum effective dose, *h* is Hill coefficient, *y* is NF-κB activity and *x* is stimulus dose. The parameters bottom, top, *h* and EC50, were optimized using the function *NonLinearModel.fit* of MATLAB R2014a (MathWorks, Natick, MA, USA).

### Sensitivity analysis

The single parameter sensitivity of each reaction is defined by

$$s_{i}(q(v),v_{i})=\frac{\partial \ln q(v)}{\partial \ln v_{i}}=\frac{\partial q(v)}{\partial v_{i}}\frac{v_{i}}{q(v)},$$

where *v*_*i*_ is an *i*th reaction, **v** is reaction vector **v**=(*v*_*1*_, *v*_*2*_, …), and *q*(**v**) is a target function, e.g., amplitude, period, Hill coefficient, EC_50_. The total sum of amplitudes of all peaks, and the average of periods were also used for the analysis. The sensitivity was calculated with 1% increases in the reaction rates.

### Numerical criteria for oscillation and switch-like response

Numerical criteria of oscillations and switch-like responses were defined as follows: oscillations have more than two peaks, which have relative amplitude values of 10^−2^ to the maximum activity. Criteria of switch-like dose responses were defined as one satisfying the following three conditions; Hill coefficient (>1), EC_50_ (<10^−4^), and dose response ratio of more than 2-fold between maximum and minimum activity values.
