# Supplementary material for: Oscillation dynamics underlie functional switching of NF-κB for B-cell activation
Source: NPJ Syst Biol Appl. 2016 Oct 20;2:16024–. doi: 10.1038/npjsba.2016.24 (PMC5516862; doi:10.1038/npjsba.2016.24)
Supplement: Supplementary Information [file npjsba201624-s1.pdf]

Supplement for “Oscillation Dynamics Underlie Functional Switching of NF-κB for B Cell Activation”

Kentaro Inoue, Hisaaki Shinohara, Marcelo Behar, Noriko Yumoto, Gouhei Tanaka, Alexander Hoffmann, Kazuyuki Aihara, Mariko Okada-Hatakeyama

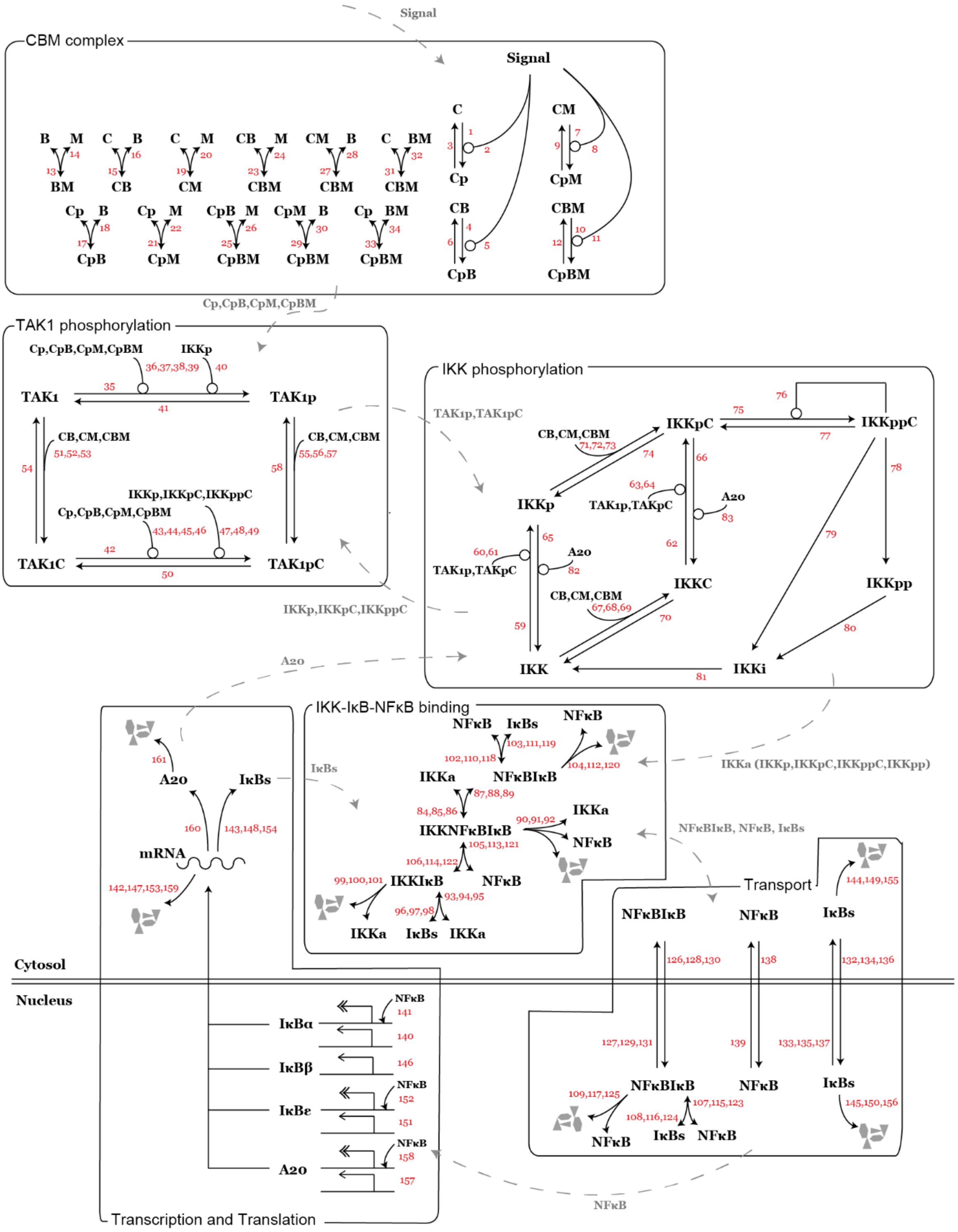

Supplementary Figure 1. Detailed diagram of the BCR- NF-κB network.

Black arrows indicate functional modifications or association/dissociation of proteins. Gray dashed arrows indicate links of inter-modules (CBM complex, TAK1 phosphorylation, IKK phosphorylation, IKK-IκB-NF-κB binding, transport, transcription and translation). There are separate components in the model for each of the three IκB isoforms (IκBα, IκBβ, IκBε). Lines with an open circle represent activation. Numbers shown on the arrows indicate reaction indices corresponding to those in **Supplementary Table 1**.

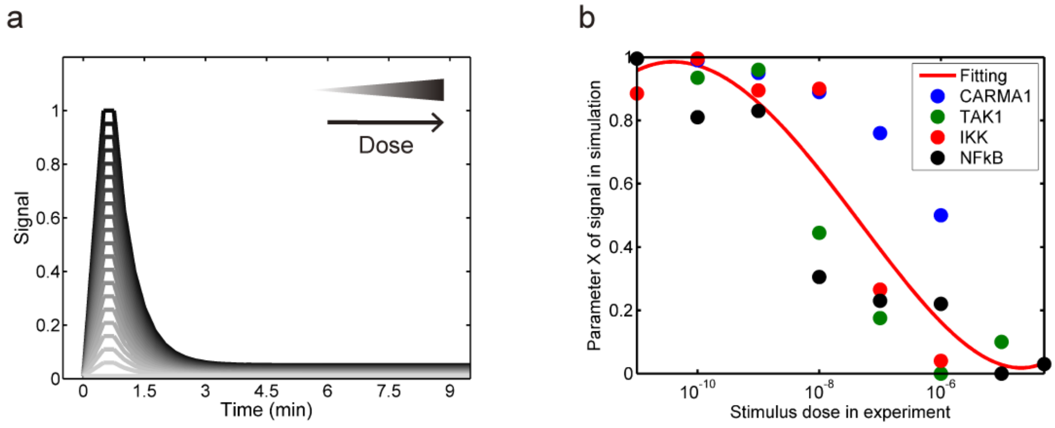

Supplementary Figure 2. Optimization for input signal using stimulus dose response experimental data.

(**a**) Dose-dependent time-course dynamics of the input signal. (**b**) Fitting the signal parameter *X* to stimulus doses in the experimental data. Filled circles indicate experimental values corresponding to the signal parameter *X* in the model. The experimental values measured dose responses of CARMA1 activity at 1 min (blue), TAK1 (green) and IKKβ (red) activity at 6 min, and nuclear NF-κB (black) at 45 min. The red line indicates a 4th order polynomial curve fitted to the experimental data.

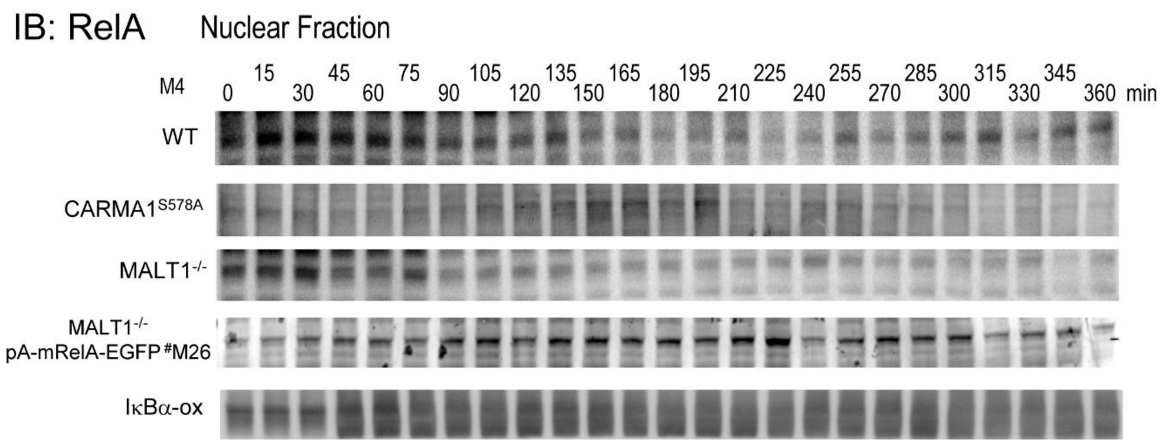

Supplementary Figure 3. Representative blots for long-term time-course of NF-κB (RelA) activity in DT40 cells.

Immunoblot with RelA antibody. Time-course activity of NF-κB was examined by the nuclear translocation of RelA. Cells were stimulated with 10 μg/ml of anti-IgM (M4) for the indicated times. Fractionated cell lysates (nuclear) were subjected to immunoblot with RelA antibody. Representative data from independent experiments (n=2~6) are shown. The quantified data of wild type (WT) are shown in **Figures 1b**, **3b** and **3g**, CARMA1 S578A mutant (CARMA1^{S578A}) in **Figure 3b**, MALT1-deficient (MALT1$^{-/-}$) in **Figure 4b** and RelA forced expression cells (RelA ox) in **Figure 4d**, and IκBα overexpression (IκBα ox) in **Figure 3g**.

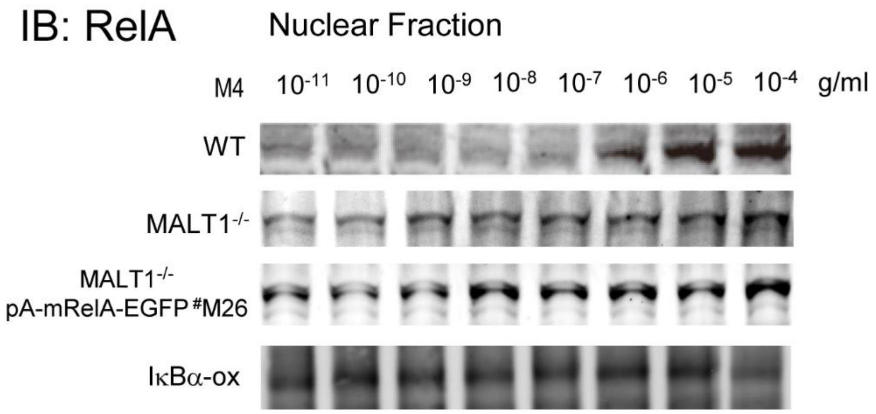

Supplementary Figure 4. Dose response of NF-κB activity.

For the dose response of NF-κB activity, cells were stimulated with the indicated anti-IgM (M4) concentrations for 45 min. Nuclear fractions were probed with RelA antibody. Representative data from independent experiments (n=2~6) are shown. The quantified data of wild type (WT) are shown in **Figure 1d**, MALT1-deficient ($MALT1^{-/-}$) in **Figure 4c** and RelA forced expression cells (RelA ox) in **Figure 4e**, and IκBα overexpression (IκBα ox) in **Figure 3i**.

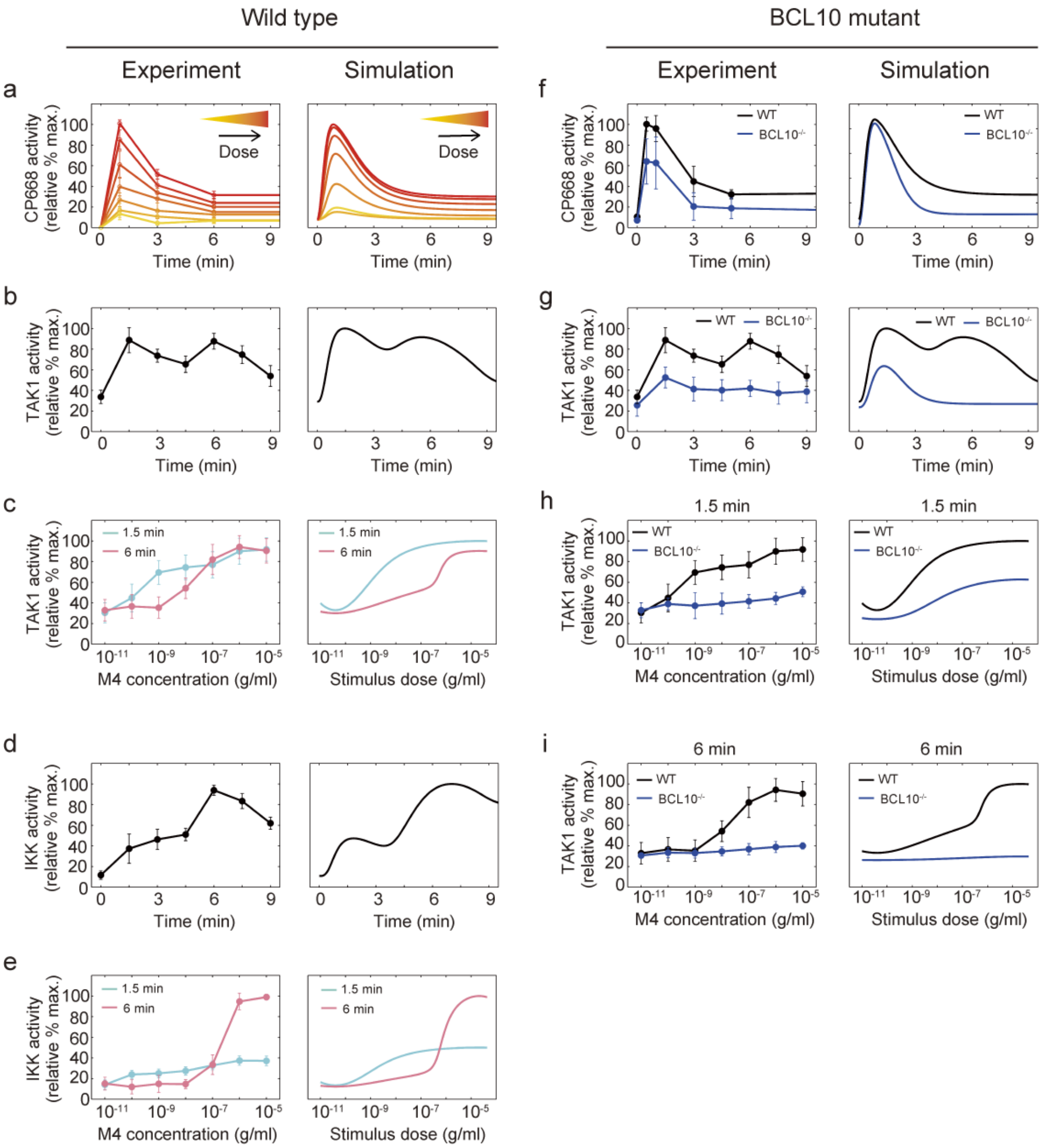

Supplementary Figure 5. Comparison of simulation results with the current model and short-term experimental data.

Simulation results obtained from the current model were compared with earlier published experiment data (*13*). (**a-e**) Wild type (WT). (**f-i**) BCL10 mutant. (**j, k**) PKC inhibitor (CGP). (**l**) IKKβ inhibitor BAY11-7085 (BAY). CGP and BAY were added at 2 min after stimulation. (**m-o**) CARMA1 ^{S578A} mutant (S578A) in simulations and previous experiments[1]. Parameters for mutants or inhibitors in the simulations are in **Supplementary Table 3**.

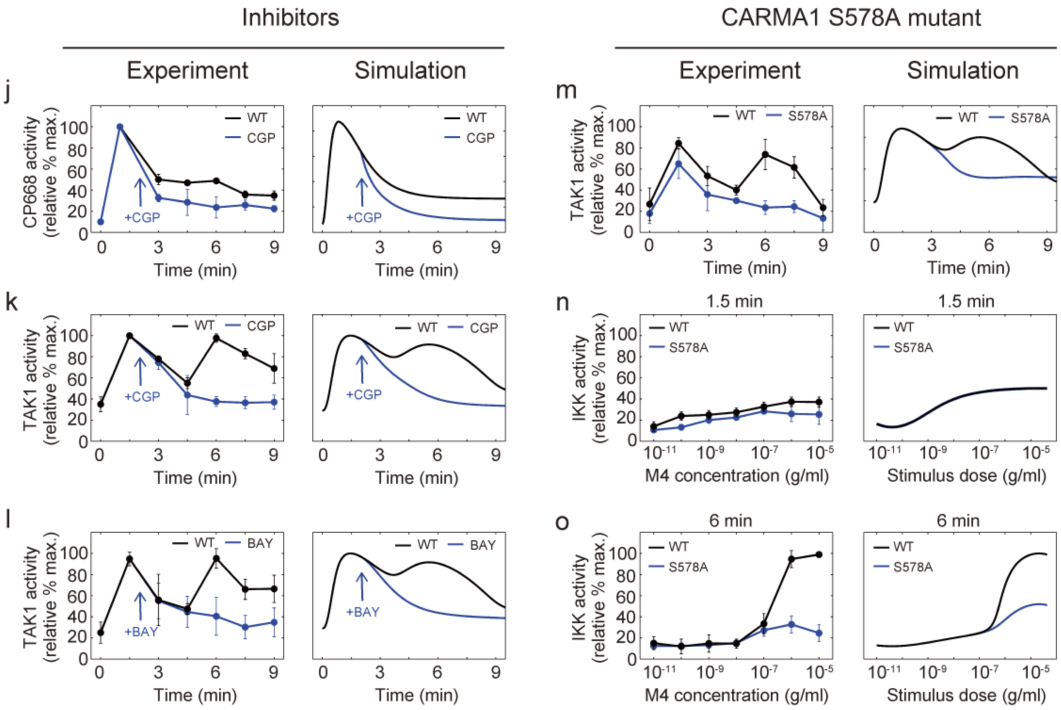

Supplementary Figure 5. Comparison of simulation results with the current model and short-term experimental data.

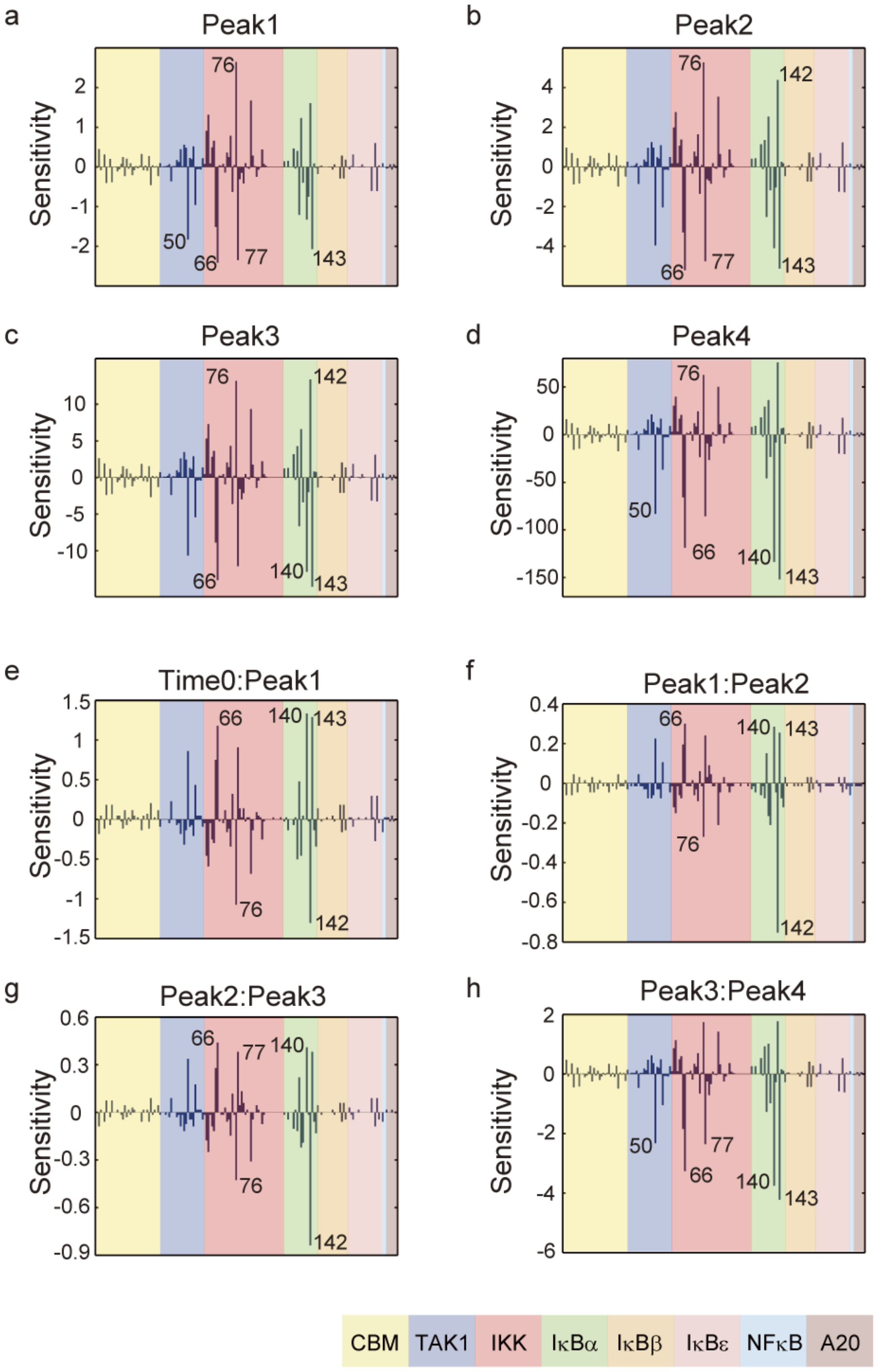

Supplementary Figure 6. Sensitivity for amplitude and period in each oscillation. Amplitudes of (**a**) the first, (**b**) second, (**c**) third and (**d**) the fourth peaks. Periods (**e**) from the stimulation to the first peak, (**f**) from the fist peak to the second peak, (**g**) from the second peak to the third peak and (**h**) from the third peak to the fourth peak. Colors indicate reaction indices for the CBM complex (yellow), TAK1 phosphorylation (blue), IKK phosphorylation (red), and IκBα (green), IκBβ (orange), IκBε (pink), NF-κB (light blue), and A20 (brown). Numbers in the figure indicate the reaction index in **Supplementary Figure 1**. Detailed sensitivity values are found in **Supplementary Table 4**.

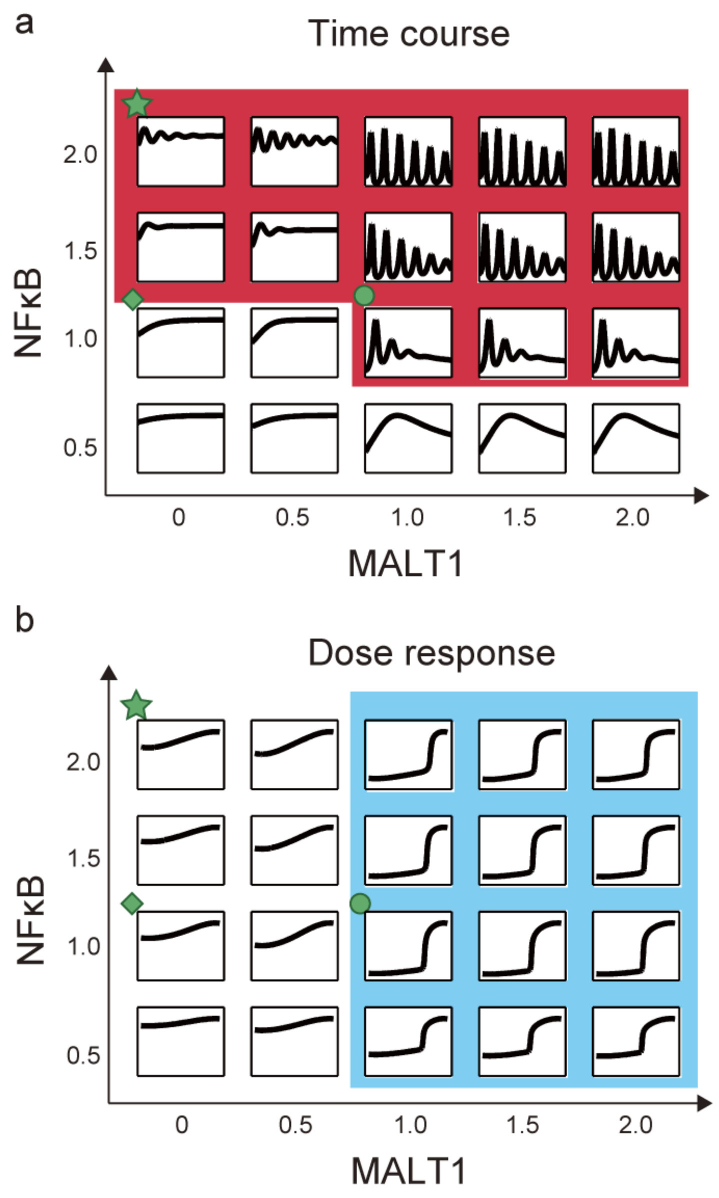

Supplementary Figure 7. Time-course and dose response dynamics of NF-κB activity with respect to combinations of MALT1 and NF-κB abundances.

(**a**) Time-course and (**b**) dose response of NF-κB activity along with the change of MALT1 and NF-κB protein abundances. Values are normalized to the maximum activity. Red indicates regions showing oscillation. Blue indicates regions showing switch-like response. Green circles are the original value. Green stars and diamonds are the parameter values experimentally validated in **Figures 4b-d**.

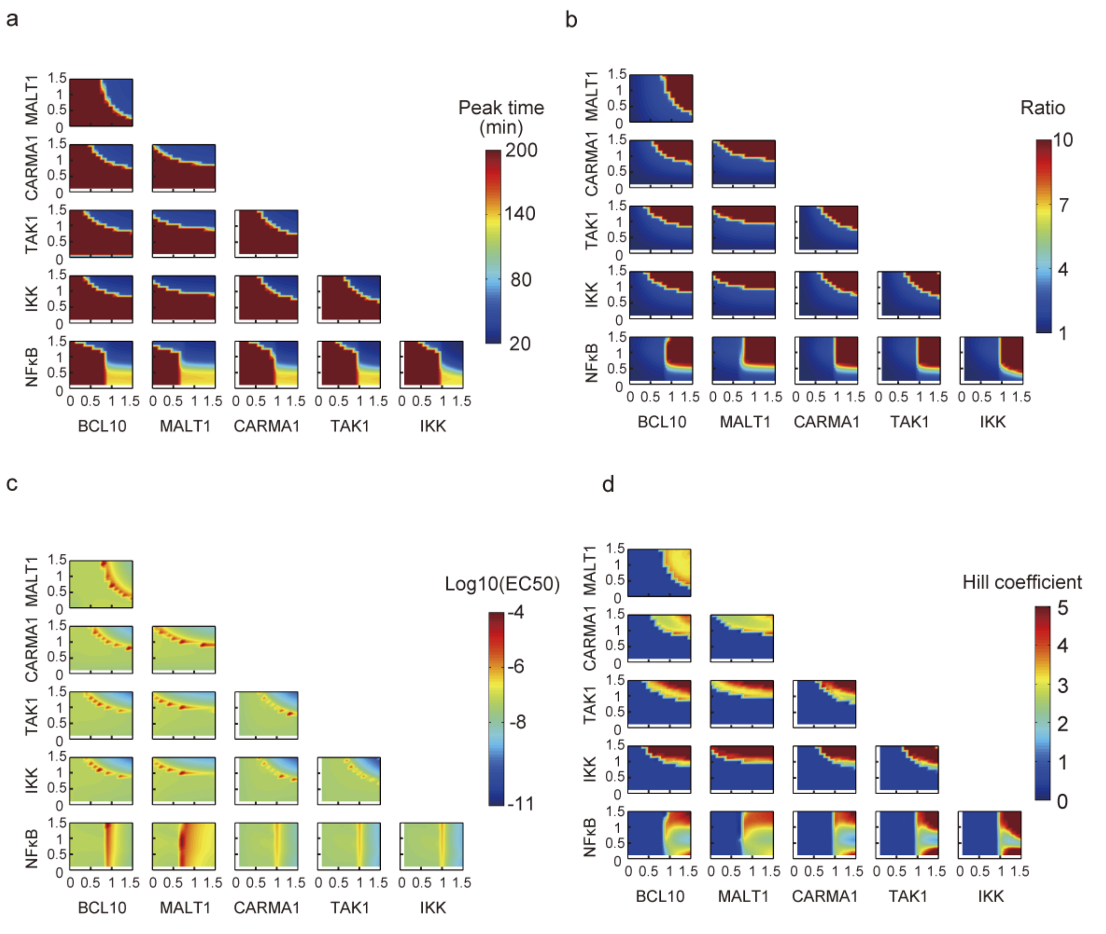

Supplementary Figure 8. Heatmaps for first peak time, ratio of maximum and minimum activity in dose response, EC_{50} and Hill coefficient.

(**a**) The first peak time in the signal parameter *X*=0. Brown indicates no peak within 360 min after stimulation. (**b**) Ratio of maximum and minimum activity in the dose response. (**c**) EC_{50}. (**d**) Hill coefficient. Large amounts of upstream signaling proteins (CARMA1, TAK1, IKKβ) result in low EC_{50} values, indicating upstream components are capable of activating NF-κB at low BCR stimuli. Hill coefficients also tend to be higher when TAK1 and IKKβ protein abundances are high. Curiously, NF-κB abundance itself has little effect on the EC_{50} or Hill coefficient (**c** and **d**) although low NF-κB abundance could not induce switch-like responses (**b** and **d**).

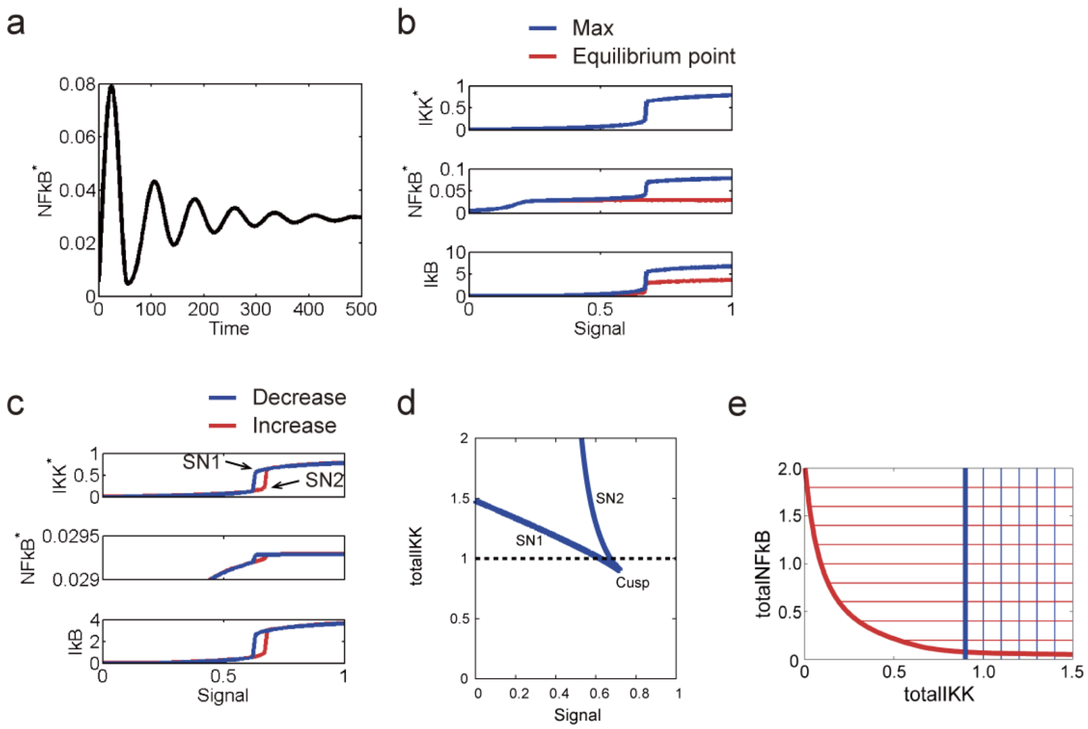

Supplementary Figure 9. Correspondence between the core model and the comprehensive model.

(**a**) Time-course of *NFkB** and (**b**) dose responses. Dose responses show the maximum and equilibrium points in the time-courses. (**c**) Equilibrium points in increase (red) or decrease (blue) of the signal dosage. (**d**) The saddle nodes (SN) and the cusp point. (**e**) Regions of oscillations (red) and switch-like responses (blue) in the core model, show qualitative correspondence to regions of oscillations and switch-like responses in the comprehensive model (**Figure 4a**).

Supplementary Table 1. Initial concentrations, rate equations, parameters and input signal function for the comprehensive model.

Supplementary Table 1 is provided as a separate excel file.

Supplementary Table 2. Value for a 4th order polynomial curve of the input signal.

Name	Value
a_1	4.052661
a_2	2.149701
a_3	3.755785×10^{-1}
a_4	2.351363×10^{-2}
a_5	4.373407×10^{-4}

Supplementary Table 3. Parameters for mutants and inhibitors in simulation.

Reaction ID	Parameter ID	Species ID	Value
		BCL10 mutant	
-	-	B	0
		PKC inhibitor	
-	slate	Signal	0.015
-	sdecay	Signal	0.25
		IKKβ inhibitor	
60	kIpTAKp	-	0
61	kIpTAKpC	-	0
63	kICpTAKp	-	0
64	kICpTAKpC	-	0
75	kIpCfaIKKpC	-	0
76	kIpCfaIKKppC	-	0
		CARMA1 S578A mutant	
47	kTpCIKK1	-	0
48	kTpCIKK2	-	0
49	kTpCIKK3	-	0
		IκBα overexpression	
140	k0mrnaikba	-	5.18×10^{-5}

Supplementary Table 4. Sensitivity for amplitude, period, Hill coefficient, EC_{50}.
Supplementary Table 4 is provided as a separate excel file.

Supplementary Table 5. Description of reactions and parameters for the core model.

index	k	km	Description of v
1	$1.00x10^{-3}$	1.8	Basal activation of IKK
2	5	0.2	Signal-dependent activation of IKK
3	10	0.4	Auto-activation of IKK
4	6	0.1	Basal inactivation of IKK
5	$8.41x10^{-4}$	1.15	Basal activation of NF-κB
6	$8.91x10^{-3}$	$5.43x10^{-1}$	IKK-dependent activation of NF-κB
7	$1.31x10^{-3}$	$2.78x10^{-3}$	IκB-dependent inactivation of NF-κB
8	$5.20x10^{-4}$	$1.79x10^{-3}$	Basal inactivation of NF-κB
9	6.76	$9.36x10^{-1}$	Basal expression of IκB
10	6.18	$7.43x10^{-5}$	NF-κB-dependent expression of IκB
11	3.66	$9.76x10^{-5}$	Basal degradation of IκB

Supplementary Table 6. Hill coefficient and EC_{50} for *CD83* mRNA expression.

Cell type	Hill coefficient	EC_{50}
Wild type	$8.19\pm5.35x10^{-16}$	$1.14x10^{-6}\pm2.93x10^{-8}$
S578A	0.567 ± 0.064	$5.936x10^{-8}\pm1.31x10^{-8}$
$MALT1^{-/-}$/RelA ox	0.362 ± 0.062	$1.65x10^{2}\pm3.86x10^{2}$
IκBα ox	0.597 ± 0.113	$9.92x10^{-7}\pm3.12x10^{-7}$

Supplementary Section 1

Description of symbols in Figure 1a

The circled letter P indicates the phosphorylated state of the amino acid residue. Lines with open circles indicate kinase phosphorylation. Arrows indicate functional modifications of the proteins or protein complexes. Double arrows indicate transcription. Filled circles indicates complexes. Blue arrows indicate positive regulation. Red lines indicate negative regulation.

Supplementary Section 2

Description of the NF-κB signaling network

We constructed a comprehensive mathematical model by integrating earlier models for BCR signaling [1] and transcriptional regulation of NF-κB [2]. We extended the models incorporating details of the formation of the CARMA1, B cell chronic lymphocytic leukemia 10 (BCL10), and/or mucosa-associated lymphoid tissue (MALT) lymphoma translocation gene 1 (MALT1) (CBM signalosome) complex, which plays critical roles in the regulation of BCR signaling (**Figure 1a** and **Supplementary Figure 1**).

BCR activation and CBM complex formation

BCR stimulation by extracellular antigen induces activation of the serine/threonine kinase protein kinase, PKCβ, which phosphorylates serine 668 (S668) of CARMA1, an adaptor protein. This modification causes a conformational change in CARMA1, allowing recruitment and activation of TAK1. Simultaneously, the phosphorylated CARMA1 recruits BCL10 and MALT1, which orchestrate the assembly of other signal modules on CARMA1. The interaction between TAK1 and IKKβ in the context of this complex causes IKKβ activation, allowing it to phosphorylate IκB, which leads to its degradation. Upon IκB degradation, NF-κB proteins accumulate in the nucleus and induce expression of target genes. This PKCβ-mediated CARMA1 (S668)-TAK1-IKKβ pathway serves as the primary axis of BCR-mediated NF-κB activation [3,4].

Positive CARMA1-TAK1-IKKβ module feedback loops in the signaling network

In a phosphorylation cascade for TAK1 and IKKβ, two positive feedback loops are operative. One is the IKKβ-dependent TAK1 activation mediated by CARMA1 phosphorylation at serine 578 (S578) [5]. Another is introduced by *trans* auto-phosphorylation of IKKβ [6]. These positive feedback regulations contribute to the switch-like activation of IKKβ and NF-κB.

Transcriptionally-inducible negative feedback loops

In resting cells, NF-κB is maintained in the cytosol in an inactive state by association with its inhibitors IκBs. There are three major IκB isoforms: IκBα, IκBβ and IκBε, which differ in their abundance and response to stimuli in terms of timing [7-10]. After stimulation, active IKKβ phosphorylates and leads to degradation of NF-κB-bound IκBs. Free NF-κB translocates into the nucleus and initiates transcription of a variety of genes, including IκBs. Newly produced IκBs then associate with NF-κB causing it to accumulate in the cytosol, thus preventing further gene regulatory activity. Expression of both IκBα and IκBε is induced

upon activation of NF-κB, although expression of IκBε is delayed [10]. TNF alpha-induced protein 3 (A20, also known as TNFAIP3) is also induced upon NF-κB activation and negatively regulates IKKβ activity [11,12].

Construction of a comprehensive mathematical model of the BCR-NF-κB network

The model considers protein interactions and modifications, and enzymatic reactions as well as protein localization. In its final form, the model is composed of 47 ordinary differential equations with 161 reactions using mass action and Michaelis-Menten kinetics.

In a resting cell, NF-κB is in an inactive state in the cytosol due to its association with inhibitor IκBs. PKCβ is activated when the B cell is activated by BCR stimulation [3]. Active PKCβ phosphorylates CARMA1 at serine 668 (S668), which is essential for NF-κB activation [3]. The phosphorylated CARMA1 at S668 (CP668) recruits BCL10 and MALT1, which enhance NF-κB activation, forming a complex (CBM complex) and phosphorylates TAK1. We assumed the four states of phosphorylated CARMA1 S668: CARMA1 phosphorylation at S668 (Cp), Cp binding BCL10 (CpB), Cp binding MALT1 (CpM), and Cp binding BCL10 and MALT1 (CpBM) because the downstream responses in BCL10 and MALT1 mutant cells after stimulation were also observed (**Figure 4** and **Supplementary Figure 5**).

TAK1 associates with the phosphorylated CARMA1 component of the CBM complex with (Cp, CpB, CpM and CpBM) and is phosphorylated. We assumed that TAK1 can exist in four states: neutral TAK1 (TAK1), TAK1 binding CBM complex (TAK1C), phosphorylated TAK1 (TAK1p), and TAK1p binding CBM complex (TAK1pC). The active forms of TAK1 are assumed to be TAK1p and TAK1pC. Active TAK1s (TAK1p and TAK1pC) phosphorylate IKK. IKK also associates with the phosphorylated CARMA1 component the CBM complex (Cp, CpB, CpM and CpBM) and is phosphorylated. We assumed that there are seven states of IKK: neutral IKK (IKK), phosphorylated IKK (IKKp), IKK in the CBM complex (IKKC), IKKp in the CBM complex (IKKpC), highly active IKK (IKKpp), IKKpp in the CBM complex (IKKppC), and inactive IKK (IKKi). The active forms of IKK are assumed as IKKp, IKKpC, IKKpp and IKKppC. Phosphorylated IKK (IKKppC) is reactivated by auto-phosphorylation [6] and phosphorylates CARMA1 at serine 578 (S578) [5]. The phosphorylated CARMA1 at S578 functions in positive feedback from IKK to TAK1 [1] and is considered implicitly in the model.

Active IKKs (IKKp, IKKpC, IKKpp and IKKppC) associate with the NF-κB and IκBs complex, and NF-κB-bound IκBs are phosphorylated, ubiquitinated and degraded by the proteasome [13]. Free NF-κB translocates into the nucleus and initiates transcription of target

genes including *IκBs* and *A20*. Expression of both IκBα and IκBε is induced upon activation of NF-κB, although expression of IκBε is delayed for 45 min [10]. Newly synthesized IκBs are found in the cytosol and nucleus and associate with free NF-κB, creating negative feedback loops [7,10,14]. A20 inhibits phosphorylation of TAK1 by IKK [11,12]. An assumption in the model is that the total protein abundance of BCL10, MALT1, CARMA1, TAK1, IKKβ and NF-κB is constant, i.e., degradation is balanced by production, then both terms are omitted in the mathematical representation.

Model parameters were fitted to recapitulate observations at two different time scales by fitting against existing short-term time-course data (up to 9 min) of TAK1, IKKβ and CARMA1 activities in wild type B cells [1] and against new long-term time-course (nuclear translocation of NF-κB was measured every 15 min up to 360 min after stimulation) and dose response (at 45 min) data of NF-κB (RelA) activity (**Figures 1b-e** and **Supplementary Figures 1-5**, **Supplementary Table 1**) using a genetic algorithm (GA).

The experiments were performed using anti-IgM (mAb M4) to stimulate chicken DT40 B cells (**Supplementary Figures 3** and **4**). We used this particular B cell line because it has a high efficiency of homologous recombination and gene targeting, thus allowing comprehensive analyses of gene regulatory networks by gene targeting mutagenesis [15]. We used a numerical function based on experimentally observed data to represent dose-dependent BCR activity (**Supplementary Figure 2**, **Supplementary Table 2**).

Optimization of input signal using stimulus dose response experimental data

The input signal is described as follows:

$$signal(t) = \begin{cases} s_{base} \quad (\text{if } 0 \le t \le t_{delay}), \\ \dfrac{s_{input} - s_{base}}{t_{raise}}(t - t_{delay}) + s_{base} \quad (\text{if } t_{delay} < t \le t_{raise} + t_{delay}), \\ s_{input} \quad (\text{if } t_{raise} + t_{delay} < t \le t_{pulse} + t_{raise} + t_{delay}), \\ (s_{input} - s_{late}) \times \exp\left(-\dfrac{t - t_{pulse} - t_{raise} - t_{delay}}{t_{decay}} \right) + s_{late} \quad (\text{if } t > t_{pulse} + t_{raise} + t_{delay}), \end{cases}$$

where s_{input}=1.0, s_{base}=0.01, s_{late}=0.05, t_{pulse}=0.25, t_{raise}=0.5, t_{decay}=0.5, and t_{delay}=0 [1]. The Hill coefficient and EC_{50} depend on the value of stimulus dose and molecular activity at that dose. To express a change in an upstream signal on different stimulus doses in the simulation, we introduced parameter *X*. Dose responses in simulation were reproduced by changing the parameter *X* at 0 to 1 as follows (**Supplementary Figure 2a**):

$$s_{input}^{'} = s_{input} - (s_{input} - s_{base}) \times X,$$

$$s_{late}^{'} = s_{late} - (s_{late} - s_{base}) \times X.$$

To optimize the input signal using stimulus dose responses derived from experiments, the experimental values in dose responses of CARMA1 activity at 1 min, TAK1 and IKKβ activities at 6 min, and nuclear NF-κB at 45 min were used. Experimental and simulation values are normalized respectively. Then, the parameter *X* of the *signal* corresponding to the experimental values (circles in **Supplementary Figure 2b**) was captured. Finally, the parameter *X* of the signal to stimulus doses in the experimental data was fitted onto a 4th order polynomial curve (**Supplementary Figure 2b, Supplementary Table 2**) as follows:

$$y = a_1 + a_2 x + a_3 x^2 + a_4 x^3 + a_5 x^4,$$

where y is the signal parameter X corresponding to stimulus dose x in the experiment.

Supplementary Section 3

Equilibrium and quasi-equilibrium analysis using the core model

The core model consists of IKKβ, NF-κB and IκB because these components showed the highest parameter sensitivities with regard to both oscillations and switch-like responses of NF-κB in our comprehensive model (**Figures 1j-m**). The equations of the core model are described as follows:

$$\frac{d[IKK^*]}{dt} = v_1 + v_2 + v_3 - v_4$$
$$= \frac{k_1(totalIKK - [IKK^*])}{km_1 + (totalIKK - [IKK^*])} + \frac{k_2 \cdot signal \cdot (totalIKK - [IKK^*])}{km_2 + (totalIKK - [IKK^*])}$$
$$+ \frac{k_3[IKK^*] \cdot (totalIKK - [IKK^*])}{km_3 + (totalIKK - [IKK^*])} - \frac{k_4[IKK^*]}{km_4 + [IKK^*]} =: F_{IKK},$$

$$\frac{d[NFkB^*]}{dt} = v_5 + v_6 - v_7 - v_8$$
$$= \frac{k_5(totalNFkB - [NFkB^*])}{km_5 + (totalNFkB - [NFkB^*])} + \frac{k_6[IKK^*] \cdot (totalNFkB - [NFkB^*])}{km_6 + (totalNFkB - [NFkB^*])}$$
$$- \frac{k_7[IkB][NFkB^*]}{km_7 + [NFkB^*]} - \frac{k_8[NFkB^*]}{km_8 + [NFkB^*]} =: F_{NFkB},$$

$$\frac{d[IkB]}{dt} = v_9 + v_{10} - v_{11} = \frac{k_9 \cdot G}{km_9 + G} + \frac{k_{10}[NFkB^*] \cdot G}{km_{10} + G} - \frac{k_{11}[IkB]}{km_{11} + [IkB]} =: F_{IkB},$$

where *IKK** is the activation form of IKKβ, *NFkB** is the activation form of NF-κB, and *IkB* is the IκB protein. The concentrations of these three forms, indicated with the square brackets, are the state variables. The reaction terms are denoted by v_i (i=1, 2,…, 11). Time evolution of the state variables is determined by the model parameters: *totalIKK*, the total abundance of *IKK*; *totalNFkB*, the total abundance of *NFkB*; *signal*, the input signal; *G*, the IκB gene; k_i and km_i (i=1, 2,…, 11), the coefficients characterizing reaction rates. The parameter values were selected so that the model can reproduce qualitative dynamics of the comprehensive model. The values of the parameters and the meanings of the reaction terms in the core model are found in **Supplementary Table 5**. In the equilibrium analysis, the value of *signal* was fixed at 1 to analyze the dynamics around an equilibrium point unless otherwise noted. The values of the other parameters were set at *totalIKK*=1, *totalNFkB*=1, and *G*=1, unless otherwise noted.

The aim of the equilibrium analysis is to show that the core model qualitatively corresponds to the comprehensive model in terms of oscillations and switch-like responses. For this purpose, we specify the conditions that the core model exhibits a damped oscillation in time course (**Supplementary Figure 9a**) and switch-like responses for variation of the signal intensity as a constant parameter (**Supplementary Figure 9b**). In the equilibrium

analysis, the onset of oscillatory dynamics, which is not sustainably periodic but damping, was characterized by the appearance of a spiral orbit around the asymptotically stable equilibrium point in the state space. By mathematically formulating such a condition and solving the conditional equations, we obtained the parameter values separating non-oscillatory and oscillatory regimes. It should be noted that the mechanism for the onset of damped oscillations is different from the typical mechanism, e.g., the Hopf bifurcation, for the onset of periodic oscillations [16]. On the other hand, the switch-like response is related to a transition from an equilibrium point to another when the signal intensity is changed. We could show that such a transition corresponds to the saddle-node bifurcation of the equilibrium point in the core model. By solving the conditional equations for the saddle-node bifurcation, we specified the parameter region where a switch-like response is observed.

First, we focus on oscillatory behavior. As a system parameter is changed, we can observe a transition between a non-oscillatory solution, which monotonically converges to an equilibrium point, and a solution with damped oscillation, which also eventually converges with a transient oscillation to an equilibrium point. The non-oscillatory solution corresponds to a non-spiral trajectory converging to a stable node in the state space, whereas the damped oscillation corresponds to a spiral trajectory converging to a stable focus in the state space. Therefore, the difference between these solutions can be characterized by the difference in the types of the vector field in the vicinity of the stable equilibrium point: for a non-oscillatory solution, the eigenvalues of the Jacobian (linearized) matrix of the core model at the equilibrium are three negative real numbers; for a damped oscillation, the eigenvalues are one negative real number and two complex conjugate numbers with a negative real part. Such a characterization is useful for finding the parameter conditions for damped oscillations [17]. The characteristic equation of the Jacobian matrix, which is satisfied by its eigenvalues λ, is written as follows:

$$\chi(\lambda) = \begin{vmatrix} \frac{\partial F_{IKK}}{\partial[IKK^*]} - \lambda & \frac{\partial F_{IKK}}{\partial[NFkB^*]} & \frac{\partial F_{IKK}}{\partial[IkB]} \\ \frac{\partial F_{NFkB}}{\partial[IKK^*]} & \frac{\partial F_{NFkB}}{\partial[NFkB^*]} - \lambda & \frac{\partial F_{NFkB}}{\partial[IkB]} \\ \frac{\partial F_{IkB}}{\partial[IKK^*]} & \frac{\partial F_{IkB}}{\partial[NFkB^*]} & \frac{\partial F_{IkB}}{\partial[IkB]} - \lambda \end{vmatrix} =: \begin{vmatrix} m_{11} - \lambda & m_{12} & m_{13} \\ m_{21} & m_{22} - \lambda & m_{23} \\ m_{31} & m_{32} & m_{33} - \lambda \end{vmatrix},$$

where the derivative components of the Jacobian matrix are denoted by m_{ij} (i=1, 2, 3 and j=1, 2, 3) for simplicity. Then, the above equation is written as follows:

$$\chi(\lambda) = -\lambda^3 - (m_{11} + m_{22} + m_{33})\lambda^2 + (m_{13}m_{31} + m_{23}m_{32} + m_{12}m_{21} - m_{11}m_{22} - m_{22}m_{33} - m_{33}m_{11})\lambda + m_{11}m_{22}m_{33} + m_{12}m_{23}m_{31} + m_{13}m_{21}m_{32} - m_{13}m_{22}m_{31} - m_{11}m_{23}m_{32} - m_{12}m_{21}m_{33} =: b_3\lambda^3 + b_2\lambda^2 + b_1\lambda + b_0,$$

where b_i is introduced to represent the coefficient of the term λ^i (i=0, 1, 2, 3). For a cubic equation like the above one, the discriminant Δ is generally given by

$$\Delta = -4b_1^3 b_3 + b_1^2 b_2^2 - 4b_0 b_2^3 + 18b_0 b_1 b_2 b_3 - 27b_0^2 b_3^2.$$

If $\Delta > 0$, the roots of $\chi(\lambda) = 0$ are three real numbers. If $\Delta < 0$, the roots are one real number and two complex conjugate numbers. Therefore, the condition $\Delta = 0$ means a transition between non-oscillatory and oscillatory solutions. Combining the equilibrium condition and the transition condition, the simultaneous equations for the boundary between non-oscillatory and oscillatory regimes of NF-κB activity are described as follows:

$$\begin{cases} F_{IKK} = 0, \\ F_{NFkB} = 0, \\ F_{IkB} = 0, \\ \Delta = 0. \end{cases}$$

By numerically solving the above equations with respect to four unknown variables including the three state variables at the equilibrium point ([*IKK**], [*NFkB**], [*IkB*]) and one system parameter, we can specify the parameter values at the onset of damped oscillation. The boundary of the oscillation region, indicated by the red curve in **Supplementary Figure 9e**, was obtained by a set of such parameter values.

Second, we turn to a switch-like response. As shown in **Supplementary Figure 9b**, the oscillation amplitude suddenly jumps as the intensity of the constant signal is increased. Our numerical investigation revealed that such a change of switch-like responses was caused by a transition from one equilibrium with a small-amplitude damped oscillation to the other, with a large-amplitude damped oscillation. The coexistence of these two equilibria is confirmed by the hysteresis in **Supplementary Figure 9c**. Numerical tracing of the lower equilibrium branch with an increase in the signal intensity showed that the lower equilibrium becomes unstable at the value indicated by SN2 due to a saddle-node bifurcation. Similarly, when the signal intensity is decreased, the upper equilibrium branch becomes unstable at the value indicated by SN1. Therefore, the existence of such saddle-node bifurcation points accounts for the switch for the maximum of *NFkB**. The saddle node bifurcation is characterized by eigenvalue 0 of the Jacobian matrix. Combining the equilibrium condition with the bifurcation condition, the simultaneous equations for the switch points are described as follows:

$$\begin{cases} F_{IKK} = 0, \\ F_{NFkB} = 0, \\ F_{IkB} = 0, \\ \chi(0) = 0. \end{cases}$$

By numerically solving the above conditions with respect to four unknown variables, including the three state variables at the equilibrium point ([*IKK**], [*NFkB**], [*IkB*]) and one system parameter (e.g. *signal*), we can specify the parameter values at SN1 and SN2. A continuation of the bifurcation points gives the bifurcation curves in the two-parameter plane as shown in **Supplementary Figure 9d**. The two saddle-node bifurcation curves are connected at the cusp point. If *totalIKK* is larger than its value at the cusp point, then the hysteretic transition, indicating a switch-like response, exists with changing the strength of the signal up and down. Since these values are almost independent of *totalNFkB*, the boundary of the parameter region for switch-like responses is obtained as the almost vertical blue line in **Supplementary Figure 9e**.

Based on the criteria for the onset of oscillatory and switch-like behaviors of NF-κB activity, we found how the oscillation region and the switch-like response region overlap (**Supplementary Figure 9e**), implying that the core model can well reproduce the essential dynamics in the comprehensive model (**Figure 4a**). That is, the switch-like activation is observed for a large value of *totalIKK*, corresponding to abundance of representative upstream molecules in the comprehensive model, and the oscillatory behavior emerged for a large value of *totalNFkB*, corresponding to high NF-κB abundance in the comprehensive model (**Figure 4a** and **Supplementary Figure 9e**).

For the quasi-equilibrium analysis, the IKK quasi-equilibrium line (or the IKK nullcline) was calculated by F_{NFkB}=0 and F_{IkB}=0, and the NF-κB quasi-equilibrium line (or the NF-κB nullcline) was calculated by F_{IKK}=0 (**Figure 5g**). To investigate the effect of the velocity for IKK activity by a positive feedback loop, we examined the dynamics for models with and without a positive feedback loop on NF-κB activity with almost the same equilibrium point. In the simulation, we increased proportionately the input signal up to 10 in 1000 min. If the velocity for IKK activity is very slow, the model trajectory would go toward the equilibrium point along with the NF-κB quasi-equilibrium line (blue dashed line in **Figure 5g**) [18]. The model trajectory with the positive feedback loop converged with more fluctuation than that without positive feedback toward the equilibrium point (**Figure 5g**). The time-course dynamics, especially the oscillation amplitude of NF-κB, were different at the orange range in **Figure 5h**.

References:

1 Shinohara, H. *et al.* Positive feedback within a kinase signaling complex functions as a switch mechanism for NF-kappaB activation. *Science* **344**, 760-764, doi:10.1126/science.1250020 (2014).

2 Werner, S. L., Barken, D. & Hoffmann, A. Stimulus specificity of gene expression programs determined by temporal control of IKK activity. *Science* **309**, 1857-1861, doi:10.1126/science.1113319 (2005).

3 Shinohara, H. & Kurosaki, T. Comprehending the complex connection between PKCbeta, TAK1, and IKK in BCR signaling. *Immunological reviews* **232**, 300-318, doi:10.1111/j.1600-065X.2009.00836.x (2009).

4 Thome, M., Charton, J. E., Pelzer, C. & Hailfinger, S. Antigen receptor signaling to NF-kappaB via CARMA1, BCL10, and MALT1. *Cold Spring Harbor perspectives in biology* **2**, a003004, doi:10.1101/cshperspect.a003004 (2010).

5 Shinohara, H., Maeda, S., Watarai, H. & Kurosaki, T. IkappaB kinase beta-induced phosphorylation of CARMA1 contributes to CARMA1 Bcl10 MALT1 complex formation in B cells. *The Journal of experimental medicine* **204**, 3285-3293, doi:10.1084/jem.20070379 (2007).

6 Polley, S. *et al.* A structural basis for IkappaB kinase 2 activation via oligomerization-dependent trans auto-phosphorylation. *PLoS biology* **11**, e1001581, doi:10.1371/journal.pbio.1001581 (2013).

7 Nelson, D. E. *et al.* Oscillations in NF-kappaB signaling control the dynamics of gene expression. *Science* **306**, 704-708, doi:10.1126/science.1099962 (2004).

8 Ashall, L. *et al.* Pulsatile stimulation determines timing and specificity of NF-kappaB-dependent transcription. *Science* **324**, 242-246, doi:10.1126/science.1164860 (2009).

9 Tay, S. *et al.* Single-cell NF-kappaB dynamics reveal digital activation and analogue information processing. *Nature* **466**, 267-271, doi:10.1038/nature09145 (2010).

10 Kearns, J. D., Basak, S., Werner, S. L., Huang, C. S. & Hoffmann, A. IkappaBepsilon provides negative feedback to control NF-kappaB oscillations, signaling dynamics, and inflammatory gene expression. *The Journal of cell biology* **173**, 659-664, doi:10.1083/jcb.200510155 (2006).

11 Skaug, B. *et al.* Direct, noncatalytic mechanism of IKK inhibition by A20. *Mol Cell* **44**, 559-571, doi:10.1016/j.molcel.2011.09.015 (2011).

12 Ma, A. & Malynn, B. A. A20: linking a complex regulator of ubiquitylation to immunity and human disease. *Nature reviews. Immunology* **12**, 774-785, doi:10.1038/nri3313 (2012).

13 DiDonato, J. A., Hayakawa, M., Rothwarf, D. M., Zandi, E. & Karin, M. A cytokine-responsive IkappaB kinase that activates the transcription factor NF-kappaB. *Nature* **388**, 548-554, doi:10.1038/41493 (1997).

14 Hoffmann, A., Levchenko, A., Scott, M. L. & Baltimore, D. The IkappaB-NF-kappaB signaling module: temporal control and selective gene activation. *Science* **298**, 1241-1245, doi:10.1126/science.1071914 (2002).

15 Shinohara, H. *et al.* PKC beta regulates BCR-mediated IKK activation by facilitating the interaction between TAK1 and CARMA1. *The Journal of experimental medicine* **202**, 1423-1431, doi:10.1084/jem.20051591 (2005).

16 Dano, S., Sorensen, P. G. & Hynne, F. Sustained oscillations in living cells. *Nature* **402**, 320-322, doi:10.1038/46329 (1999).

17 Zambrano, S., Bianchi, M. E. & Agresti, A. A simple model of NF-kappaB dynamics reproduces experimental observations. *J Theor Biol* **347C**, 44-53, doi:10.1016/j.jtbi.2014.01.015 (2014).

18 Behar, M., Barken, D., Werner, S. L. & Hoffmann, A. The dynamics of signaling as a pharmacological target. *Cell* **155**, 448-461, doi:10.1016/j.cell.2013.09.018 (2013).
